# Analysis of the *Fgfr2*^C342Y^ mouse model shows condensation defects due to misregulation of *Sox9* expression in prechondrocytic mesenchyme

**DOI:** 10.1242/bio.022178

**Published:** 2017-01-09

**Authors:** Emma Peskett, Samin Kumar, William Baird, Janhvi Jaiswal, Ming Li, Priyanca Patel, Jonathan A. Britto, Erwin Pauws

**Affiliations:** 1 UCL Great Ormond Street, Institute of Child Health, University College London, London, WC1N 1EH, UK; 2 Craniofacial Unit, Great Ormond Street Hospital, London, WC1N 3JH, UK

**Keywords:** Crouzon, Craniosynostosis, FGFR2, Mesenchyme, SOX9, RUNX2

## Abstract

Syndromic craniosynostosis caused by mutations in *FGFR2* is characterised by developmental pathology in both endochondral and membranous skeletogenesis. Detailed phenotypic characterisation of features in the membranous calvarium, the endochondral cranial base and other structures in the axial and appendicular skeleton has not been performed at embryonic stages. We investigated bone development in the Crouzon mouse model (*Fgfr2*^C342Y^) at pre- and post-ossification stages to improve understanding of the underlying pathogenesis. Phenotypic analysis was performed by whole-mount skeletal staining (Alcian Blue/Alizarin Red) and histological staining of sections of CD1 wild-type (WT), *Fgfr2*^C342Y/+^ heterozygous (HET) and *Fgfr2*^C342Y/C342Y^ homozygous (HOM) mouse embryos from embryonic day (E)12.5-E17.5 stages. Gene expression (*Sox9*, *Shh*, *Fgf10* and *Runx2*) was studied by *in situ* hybridisation and protein expression (COL2A1) by immunohistochemistry. Our analysis has identified severely decreased osteogenesis in parts of the craniofacial skeleton together with increased chondrogenesis in parts of the endochondral and cartilaginous skeleton in HOM embryos. The *Sox9* expression domain in tracheal and basi-cranial chondrocytic precursors at E13.5 in HOM embryos is increased and expanded, correlating with the phenotypic observations which suggest FGFR2 signalling regulates *Sox9* expression. Combined with abnormal staining of type II collagen in pre-chondrocytic mesenchyme, this is indicative of a mesenchymal condensation defect. An expanded spectrum of phenotypic features observed in the *Fgfr2*^C342Y/C342Y^ mouse embryo paves the way towards better understanding the clinical attributes of human Crouzon–Pfeiffer syndrome. *FGFR2* mutation results in impaired skeletogenesis; however, our findings suggest that many phenotypic aberrations stem from a primary failure of pre-chondrogenic/osteogenic mesenchymal condensation and link FGFR2 to SOX9, a principal regulator of skeletogenesis.

## Abstract (precis)

**Summary:** Mutation of FGFR2 causes a misregulation of *Sox9*, leading to disrupted mesenchymal condensation, and thus skeletal and craniofacial birth defects in mice, with implications for human Crouzon syndrome.

## INTRODUCTION

Syndromic craniosynostosis can be caused by mutations in the *FGFR2* gene and is inherited in an autosomal dominant manner (Wilkie, 2005). One of the most common syndromes is Crouzon syndrome, where patients are characterised by coronal craniosynostosis, midfacial hypoplasia and proptosis, generally without limb defects (Reardon et al., 1994). More severely affected patients, especially those with limb defects, are often described as Pfeiffer syndrome (Rutland et al., 1995). Together with rarer conditions such as Jackson–Weiss and Beare–Steveson syndrome, these patients are clinically and genetically assumed to be part of the same phenotypic spectrum as they can share gain-of-function *FGFR2* mutations and are often referred to as Crouzon–Pfeiffer syndrome (CPS). Less common features include hearing loss, tracheal cartilaginous sleeve, butterfly vertebrae and cleft palate (Helman et al., 2014). Some of these features can also be seen in patients with Apert syndrome (AS), which is also caused by mutations in *FGFR2* (Wilkie et al., 1995).

The fibroblast growth factor (FGF) signalling pathway is activated by extracellular FGF ligands that bind to the extracellular domain of FGF receptors causing intracellular signal transduction. FGF signalling­-regulated gene transcription has been associated with pre- and postnatal growth. During embryonic development it regulates proliferation, cell survival, differentiation and migration, while in adult tissues it is involved with homeostasis and regeneration (Ornitz and Itoh, 2001). The most common mutation in FGFR2 that causes CPS affects cysteine 342. This amino acid is located in the third Ig-loop (IgIII) of the extracellular part of the FGF receptor and is specific to the FGFR2c isoform, which plays an important role in the embryonic development of the (craniofacial) skeleton (Eswarakumar et al., 2002). Previously, a mouse knock-in of the human C342Y mutation (i.e. *Fgfr2*^C342Y^) was found to mimic human Crouzon syndrome with many of the clinical features present including coronal craniosynostosis (Eswarakumar et al., 2004). These studies have focussed on the craniofacial features that involve sutural fusion of intramembranous bones of the calvarium, and have suggested a role for FGFR2 in the balance between proliferation and differentiation of sutural mesenchyme. In addition they have shown that inhibition of FGFR signalling can attenuate phenotypic features (Eswarakumar et al., 2006). Mutation of FGFR2 has been associated with hyperactivation of the RAS-ERK pathway in Crouzon (Pfaff et al., 2016) and Apert (Wang et al., 2010) mouse models. Elsewhere it has been shown that the initial patterning of the coronal suture during mouse embryonic development around embryonic day (E)11.0 relies on correct expression of *En1*, which in turn regulates the correct expression of *Fgfr2* and the onset of osteogenic differentiation (Deckelbaum et al., 2012).

Contrary to intramembranous bone formation in the calvaria, most of the bones in the cranial base and most bones of the axial skeleton are formed through endochondral ossification. *FGFR2* has been shown to be expressed throughout the human embryonic membranous calvarium, sutural mesenchyme as well as the endochondral skull base (Britto et al., 2001), and the human embryonic palatal medial edge epithelium (Britto et al., 2002). Endochondral bone formation is characteristically preceded by a cartilage anlage formed through chondrocytic differentiation of the mesenchyme, followed by the invasion and differentiation of osteoblasts replacing the cartilage with bone (Zelzer and Olsen, 2003). The early stages of pre-cartilaginous mesenchymal condensation, as well as the differentiation of chondrocytes into mature cartilage, are known to be regulated by SOX9 (De Crombrugghe et al., 2000). Other skeletal structures are entirely made of cartilage that does not transform into bone and these can also be affected in patients with CPS. C-shaped cartilage rings situated on the ventral and lateral side of the trachea provide structural support while keeping it flexible. During the embryonic development of the trachea, *Fgf10* is expressed in the ventral, pre-chondrocytic mesenchyme and inactivation as well as overexpression of *Fgf10* cause abnormal patterning of cartilage rings. FGF10, through its receptor FGFR2b, regulates the segmented expression of *Shh*, which is responsible for the pre-cartilaginous condensation of ring structures (Sala et al., 2011). As such, inactivation of *Shh* leads to a complete lack of tracheal cartilage due to a downregulation of *Sox9* expression (Park et al., 2010). *Sox9* is expressed in undifferentiated mesenchyme where it is involved in the condensation of pre-chondrocytic structures as well as the differentiation and maturation of chondrocytic cartilage (Elluru and Whitsett, 2004; Hall and Miyake, 2000). Chondrocytic differentiation requires extracellular matrix (ECM) organization. Type II collagen (COL2A1) is an important component of cartilage ECM and is directly regulated by SOX9 (Lefebvre and de Crombrugghe, 1998). A link between FGFR2 and *Sox9* has also been established in the development of the pancreas (Seymour et al., 2012a) and the testis (Bagheri-Fam et al., 2008). It has been shown that induction of FGF-FGFR signalling increases *Sox9* levels *in vitro* (Murakami et al., 2000a). Therefore, and because *Sox9* is essential for normal cartilage formation (Bi et al., 1999), it is a good candidate downstream target of mutant FGFR2 in the pathogenesis of chondrocytic defects in CPS.

This study focusses on the phenotypic spectrum of homozygous embryos at different stages of development in an attempt to elucidate the molecular and cellular mechanisms behind CPS caused by FGFR2 mutation. We hypothesize that the homozygous mutant will be a more severe version of the heterozygote and make it easier to study molecular events at embryonic stages, before the onset of the skeletal phenotype. Detailed analysis of the Crouzon mouse model at embryonic stages showed all known features as reported in the literature, and in addition identified some previously unreported phenotypic features, particularly in the homozygous mutants. Homozygous embryos do not survive birth, mainly due to the cleft palate phenotype, but as they represent the most severe end of the clinical spectrum of human CPS, and to a certain extent of AS, they can be of great value when trying to clarify the role of FGFR2 in the pathogenesis of these birth defects.

## RESULTS

### Homozygous mutation of FGFR2 causes exencephaly

**Fig. 1.**
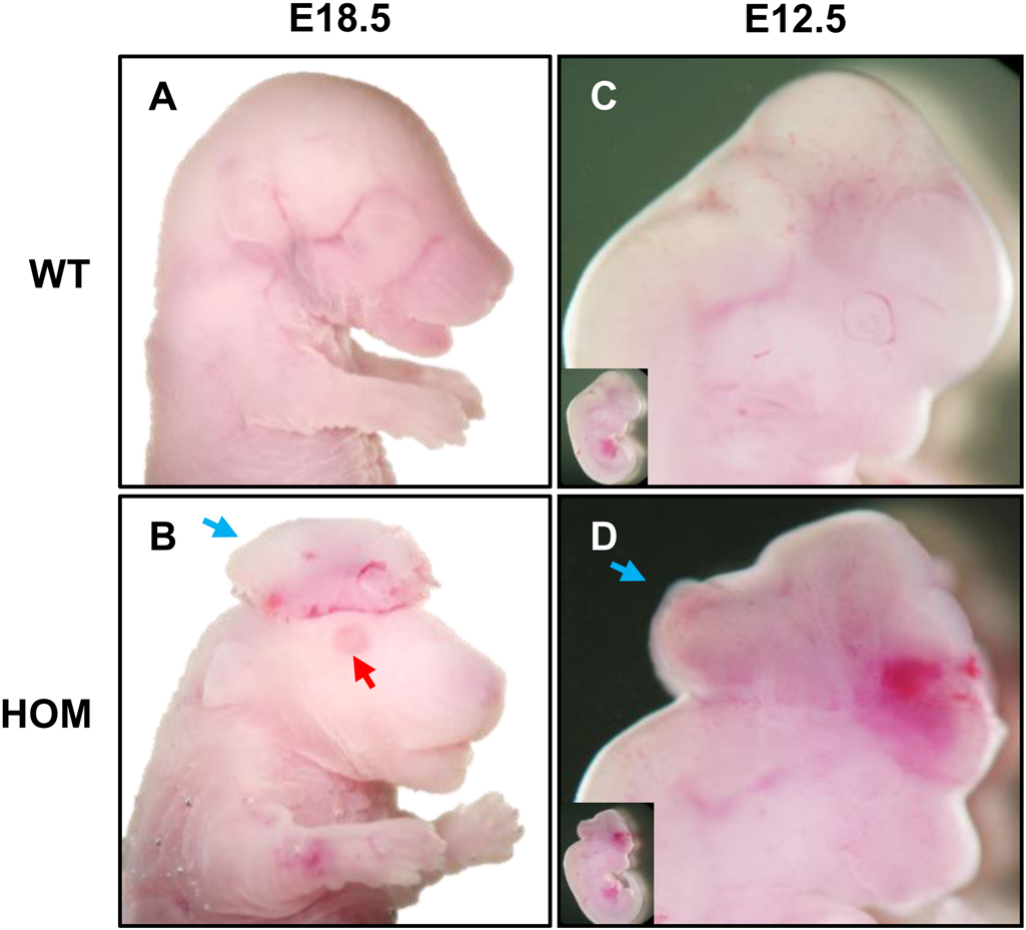
**Exencephaly phenotype in FGFR2-C324Y homozygous embryos.** (A,C) Normal development in wild-type embryos at E18.5 (A) and E12.5 (C). (B) Approximately 63% (*n*=12/19) of FGFR2-C324Y homozygous (HOM) mutants display exencephaly (blue arrow) at E18.5, often together with absent eye lids (red arrow). (D) Signs of exencephaly can be observed as early as E12.5 in HOM mutants (58%, *n*=14/24), well before the start of calvarial bone development.

**Fig. 2.**
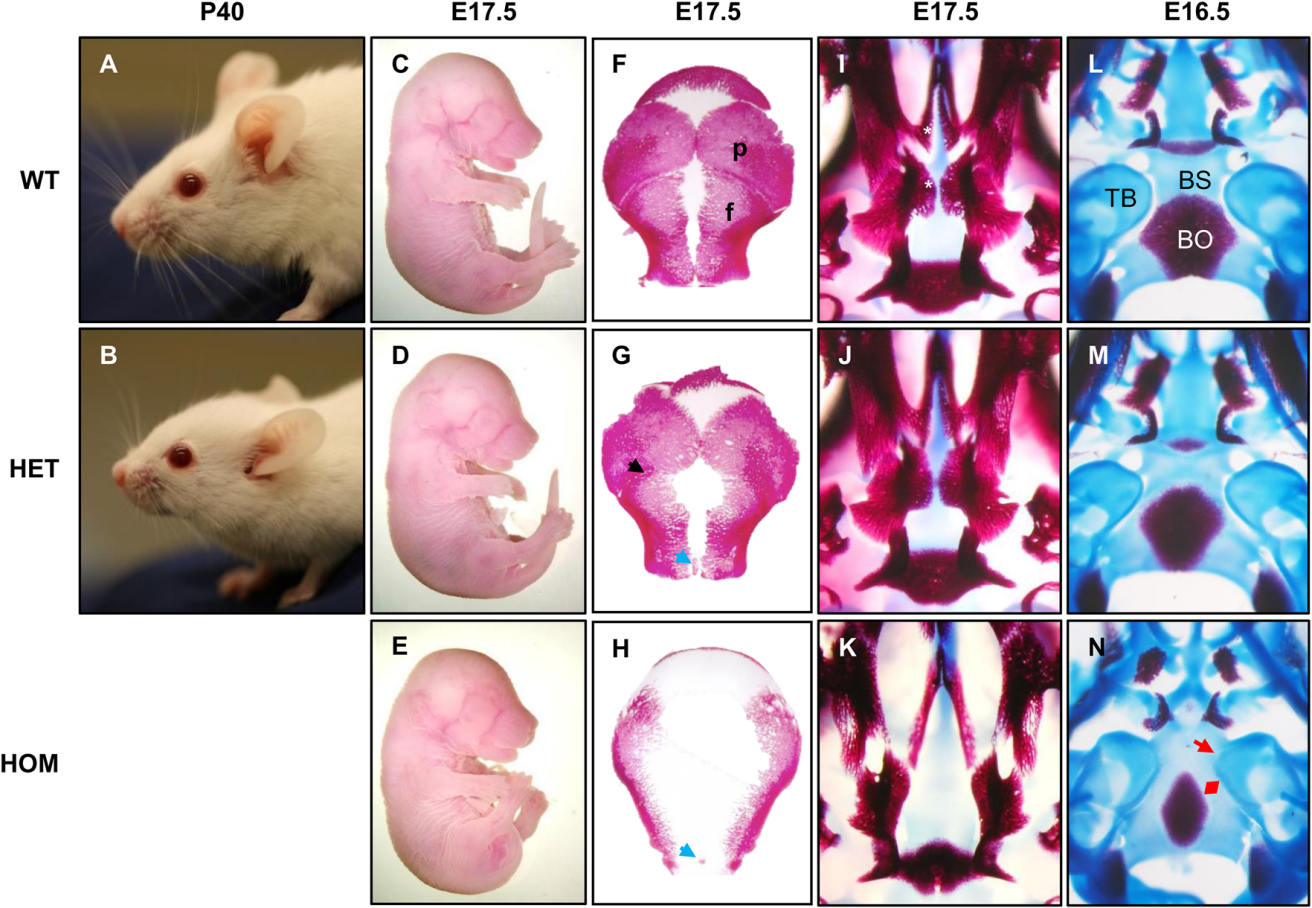
**Analysis of the craniofacial skeletal phenotypes of the FGFR2-C342Y Crouzon mouse model.** (A,B) Craniofacial appearance of *Fgfr2*^+/+^ (WT) and *Fgfr2*^C342Y/+^ (HET) mice at postnatal day (P)40. Note the brachycephaly and midfacial hypoplasia (short snout). Homozygous mutant *Fgfr2*^C342Y/C342Y^ (HOM) animals die postnatally due to cleft palate. (C-E) Gross morphology of E17.5 embryos. Homozygous embryos are smaller with a more rounded head shape. (F-H) Dorsal view of the calvarial bones stained with Alizarin Red. Heterozygous embryos show partially fused coronal sutures (black arrow) at this stage. Homozygotes have no coronal sutures and display bone hypoplasia. An interfrontal Wormian bone (blue arrows) can be observed in both mutants. (I-K) Ventral view of the anterior skull stained with Alizarin Red and Alcian Blue. Palatal bones in WT are indicated by asterisks. Homozygous embryos show cleft palate. (L-N) Ventral view of the posterior skull including the cranial base. Homozygous embryos show fusion of the tympanic bulla and the sphenoid-occipital bone (red arrow). Ossification of the occipital (red double arrow) and other bones is hypoplastic. Heterozygotes show a partially penetrant ossification phenotype, without tympanic-sphenoid-occipital fusion. All experiments were performed on at least three embryos (*n*=3) for each genotype. f, frontal bone; p, parietal bone; TB, tympanic bulla; BS, basisphenoid; BO, basioccipital.

**Fig. 3.**
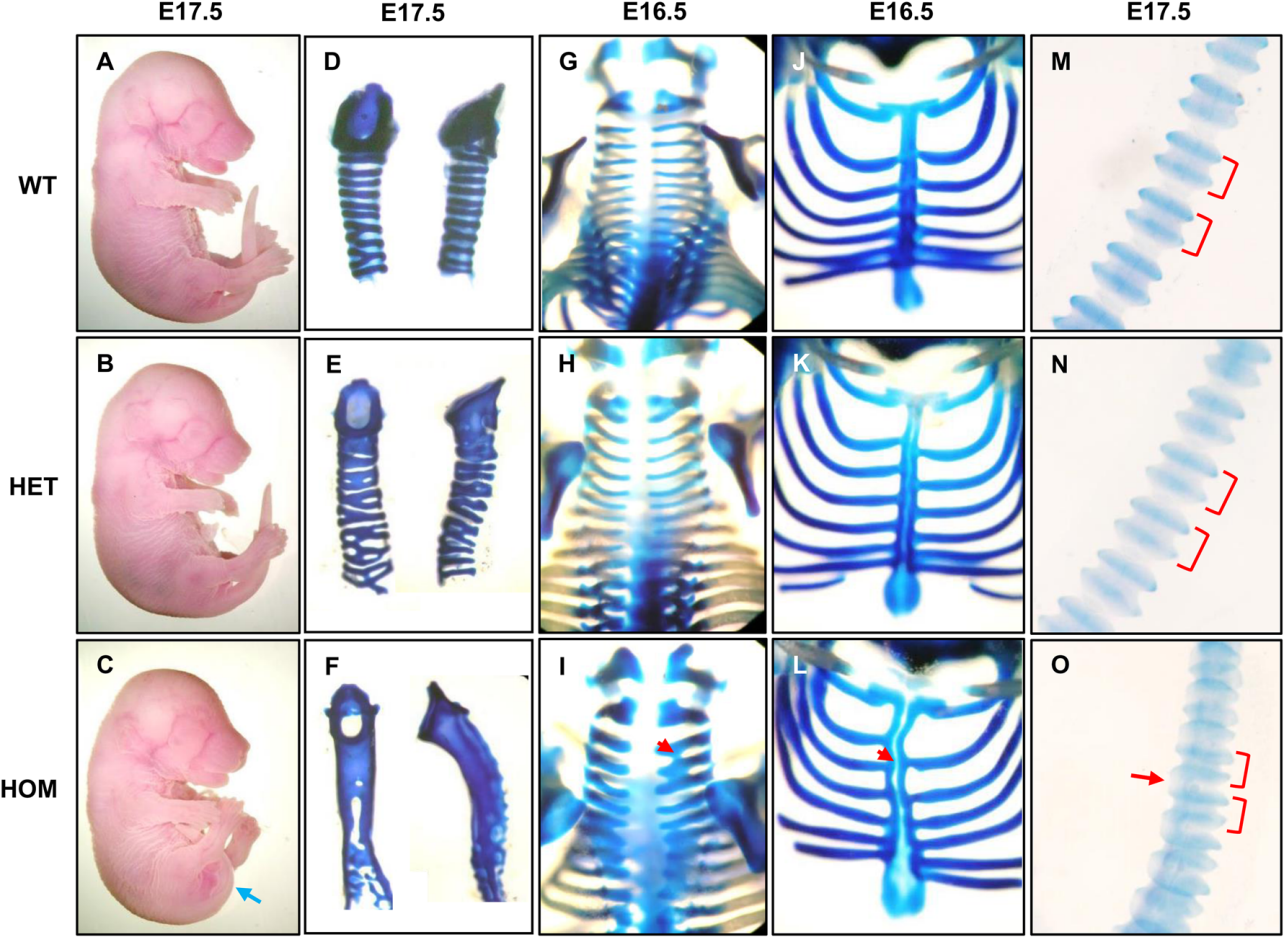
**Analysis of the axial/appendicular skeletal phenotype of the FGFR2-C342Y Crouzon mouse model.** (A-C) Gross morphology of E17.5 embryos. Homozygous embryos display a curved tail phenotype (blue arrow). (D-F )Trachea stained with Alcian Blue. Homozygotes show a cartilaginous sleeve phenotype, heterozygous mutants display a partially penetrant phenotype with variable inter-ring fusion focussed at the proximal/rostral end. (G-I) Cervical vertebrae show thickening and fusion (red arrow) of cartilaginous structures in homozygous embryos only. (J-L) Rib cage disorganisation and cleft sternum (red arrow) can be observed in homozygous embryos with heterozygous embryos showing an intermediate, less severe phenotype. (M-O) The curvature of the tail in homozygous mutants is increased, and the tail is generally shorter compared to controls (see A-C). Staining of cartilaginous tail shows caudal vertebrae to be closer together (red brackets) and an inter-vertebral bridge (red arrow) has formed on the ventral side. All experiments were performed on at least three embryos (*n*=3) for each genotype.

Neural tube defects (NTD) have not been reported in human cases of CPS. However, in our hands, approximately 50% of embryos homozygous for the *Fgfr2*^C342Y^ mutation display exencephaly (Fig. 1B,D). The protruding brain can be seen as early at E12.5 (Fig. 1D), which is well before the development of calvarial bones, excluding the option that this is a secondary feature of the cranial bone defects. A minority of embryos (<1%) also show spina bifida or complete cleft face (data not shown). In addition, we found that the tail of homozygous embryos is shorter and curved abnormally towards the ventral trunk (Fig. 2E), a feature associated with mouse models of spina bifida. Analysis of the cartilaginous vertebrae in the distal tail shows fusion on the ventral side (Fig. 3M-O), which would explain the direction of the abnormal tail curvature, but seems to exclude a caudal neural tube defect. The exencephaly phenotype prevents complete analysis of the craniofacial skeleton, but increased levels of skeletal hypoplasia elsewhere, as well as the frequent observation that eyelids are missing in these embryos (Fig. 1B) makes it likely that these are at the most severe end of the phenotypic spectrum.

### Homozygous mutation of FGFR2 causes cranial base dysmorphology

To analyse the pre-synostosis craniofacial phenotype in *Fgfr2*^C342Y^ mutant embryos we stained bone (Alizarin Red) and cartilage (Alcian Blue) in skulls collected between E15.5 and 17.5 (Fig. 2). As previously reported, homozygous mutants have cleft palate with full penetrance (Fig. 2I-K). The first signs of coronal synostosis can be seen from E17.5 onwards in heterozygotes while homozygotes don't appear to have a coronal suture at this stage (Fig. 2F-H). Calvarial bones are significantly smaller in homozygous mutants, with signs of hypo-ossification in heterozygous embryos too. Closer inspection of the calvaria shows that all mutants –heterozygotes and homozygotes – have a small Wormian bone located between the anterior frontal bones (Fig. 2G,H). This interfrontal bone is common in some wild-type strains but rare in CD1 mice and is absent from wild-type controls. The cranial base of the homozygous E16.5 embryo shows fusion of the cranial base (occipital-sphenoid bone) and the bony part of the inner ear (tympanic bulla) (Fig. 2L-N). This results in an abnormal shape of the inner ear and cochlea, which may be a contributing factor to the many reasons for hearing impairment reported in cases of CPS. Furthermore, osteogenic hypoplasia can be observed by the lower levels of ossification in the cranial base as well as the calvarium shown by Alizarin Red staining in homozygous mutants, with heterozygous mutants presenting an intermediate phenotype.

### Homozygous mutation of FGFR2 causes pronounced defects in the axial skeleton

Phenotypic features in the axial skeleton (Fig. 3) include defects in cartilaginous structures, which will become either endochondral bone or cartilage. Tracheal cartilaginous sleeve, which is a rare finding in the most severely affected CPS patients, is found in all homozygous mutants (Fig. 3D-F). Heterozygous mutants display a hypomorphic, asymptomatic phenotype, with partial fusion of cartilage rings present mainly at the proximal part of the trachea including fusion of the cricoid to the first tracheal ring, while thyroid and hyoid cartilages appear normal (data not shown). At the rostral end of the vertebral column, thickening and partial fusion of cervical vertebrae can be observed exclusively in the homozygous mutants (Fig. 3G-I), which may be reminiscent of ‘butterfly’ vertebrae in CPS patients. Both heterozygous and homozygous mutants present with rib cage abnormalities and cleft sternum, with homozygous mutants displaying the more severe defects (Fig. 3J-L).

### Dysregulation of FGFR2 signalling increases *Sox9* expression in prechondrocytes

**Fig. 4.**
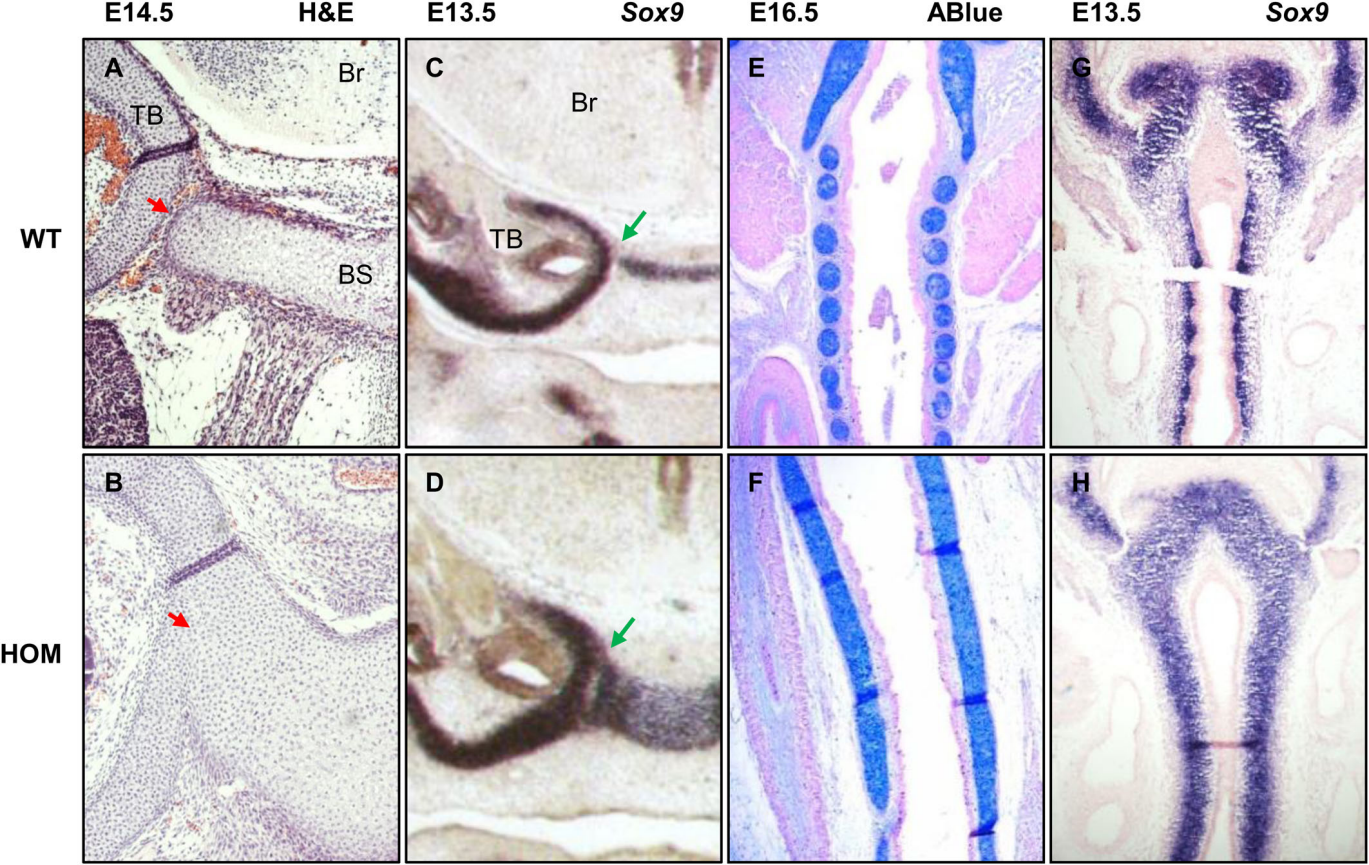
**Histological and expression analysis of cranial base and trachea at different embryonic stages.** (A,B) Coronal sections through the tympanic bulla (TB) and basisphenoid (BS) were stained with Haematoxylin and Eosin. Fusion can be observed in mutant (HOM) E14.5 embryos. Red arrows show fusion between the cranial base and the inner ear mesenchyme. (C,D) *Sox9 in situ* hybridisation (ISH) at E13.5 on cranial base sections shows increased and ectopic expression. Green arrows show the area between the cranial base and the inner ear mesenchyme. (E,F) Coronal sections through the trachea at E16.5 stained with Alcian Blue show fused cartilage rings in HOM embryos. (G,H) *Sox9* ISH at E13.5 on trachea shows expression in mutant embryos is increased and ectopic. Only WT embryos show condensation of cartilage precursors into ring structures. Br, brain; TB, tympanic bulla; BS, basisphenoid. All experiments were performed on at least three embryos (*n*=3) for each genotype.

During cartilage formation *Sox9* is both a marker of chondrocyte progenitors and chondrocyte maturation. To establish whether FGFR2 mutation affects *Sox9* expression during embryonic stages of cartilage development we performed a more detailed analysis focussing on the cranial base and the trachea (Fig. 4). Fusion between the cranial base and the inner ear mesenchyme (Fig. 4A,B), and of the proximal tracheal rings (Fig. 4E,F) can be observed histologically at a stage prior to the onset of chondrogenic maturation. Analysis of *Sox9*, an early marker of chondrocyte precursors, shows increased and ectopic expression in *Fgfr2*^C342Y^ homozygous mutants. In the cranial base, separate condensation of the sphenoid and the bulla seems to be prevented by an enlarged expression domain of Sox9 (Fig. 4C,D). In the wild-type trachea at E13.5, *Sox9* expression exhibits a segmented pattern that forms the basis for the organisation into separate cartilage rings, while the mutant displays an enlarged expression domain in the trachea and cricoid but not in the upper cartilages and lungs (data not shown) preventing segmentation (Fig. 4G,H).

### FGFR2­-related upregulation of SOX9 causes mesenchymal condensation defects

**Fig. 5.**
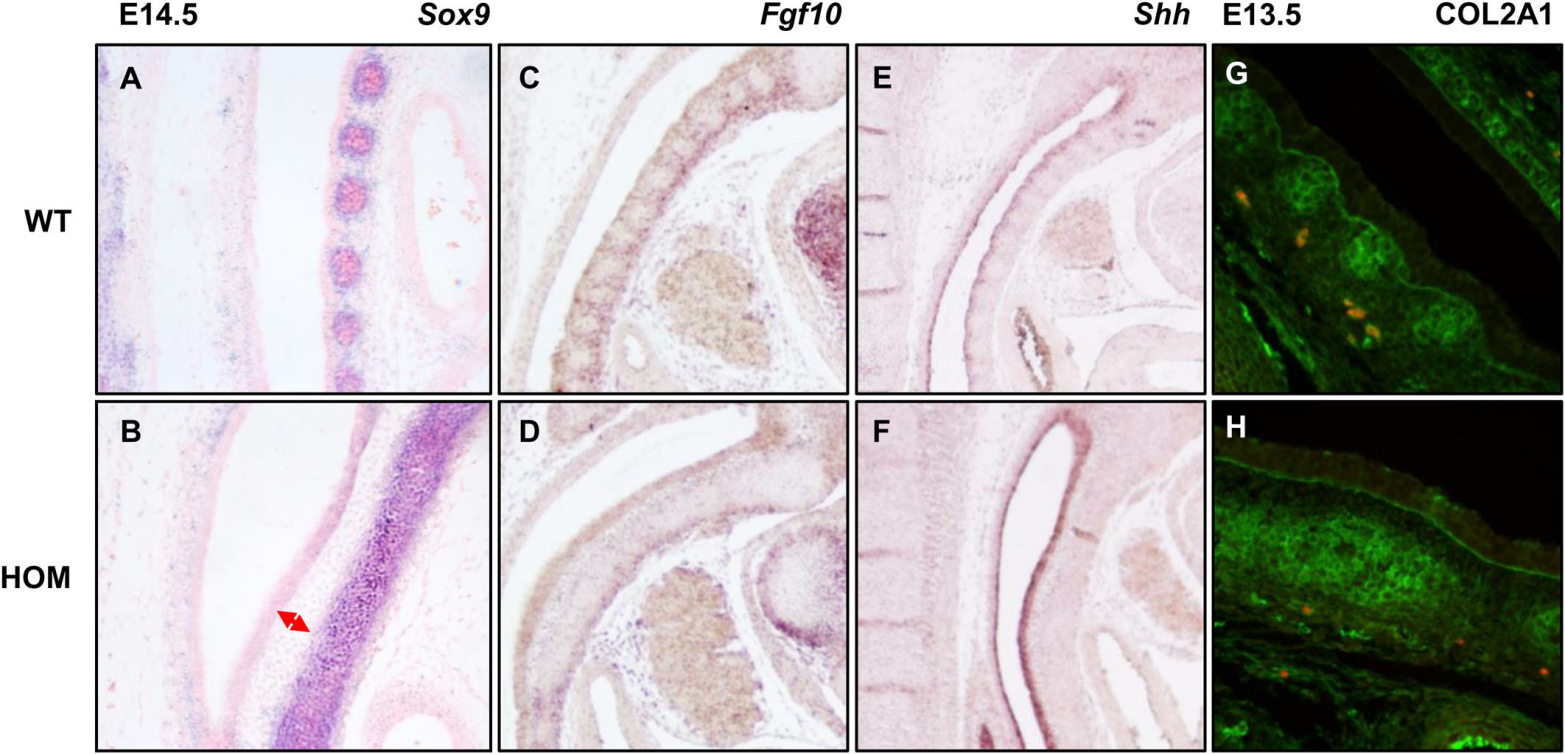
**Expression analysis during tracheal ring development.** (A,B) At E14.5 *Sox9* expression in the WT is now restricted to the outer edge of pre-cartilaginous condensations. In the HOM mutant expression is persistent throughout an unsegmented sheet with a distinct gap (red double arrow) between the condensation and the epithelium. (C,D) *Fgf10* expression in the HOM mutant at E13.5 is decreased and inter-segmental expression between pre-cartilaginous condensations is lost. (E,F) *Shh* expression at E13.5 is increased in the dorsal epithelium but normal in other areas, including the ventral epithelium. (G,H) At E13.5 COL2A1 expression by immunohistochemistry shows a thickened pre-cartilaginous condensation lacking segmentation. This emphasizes the gap between condensed mesenchyme and dorsal epithelium which causes a loss of contact between the chondrocytic mesenchyme and the basal side of the epithelium. All experiments were performed on at least three embryos (*n*=3) for each genotype.

An additional observation when analysing the expression domain of *Sox9* in homozygous *Fgfr2*^C342Y^ mutants at E13.5, well before cartilage maturation, was the presence of a distinct gap between the *Sox9*-positive mesenchyme and the epithelium, contrary to the wild-type where *Sox9*-positive cells are aligned along the basal surface (Fig. 5A,B). Two main determinants of tracheal ring formation are *Fgf10* and *Shh*. In *Fgfr2* homozygous mutants, *Shh* expression in the ventral epithelium appears increased against the wild-type control and also compared to the dorsal epithelium (Fig. 5E,F). This may be a compensatory mechanism as a result of a failure of SHH to reach its target cells due to the inter-epithelial-mesenchymal gap. Also, expression of *Fgf10* appears decreased at the ventral side of the trachea, and is absent from the non-chondrocytic mesenchyme separating the primordial tracheal rings (Fig. 5C,D). Finally, analysis of COL2A1 – a target of SOX9 during cartilage maturation – in the extracellular matrix (ECM) of tracheal mesenchyme shows an expanded expression domain in the homozygous mutant that corresponds to the *Sox9* expression data (Fig. 5G,H). In addition, no COL2A1 is present in the gap between the epithelium and mesenchyme, suggesting that the chondrocytic ECM in the tracheal mesenchyme is detached from the basal side of the tracheal epithelium.

### FGFR2-C342Y homozygous mutants do not form a coronal suture

**Fig. 6.**
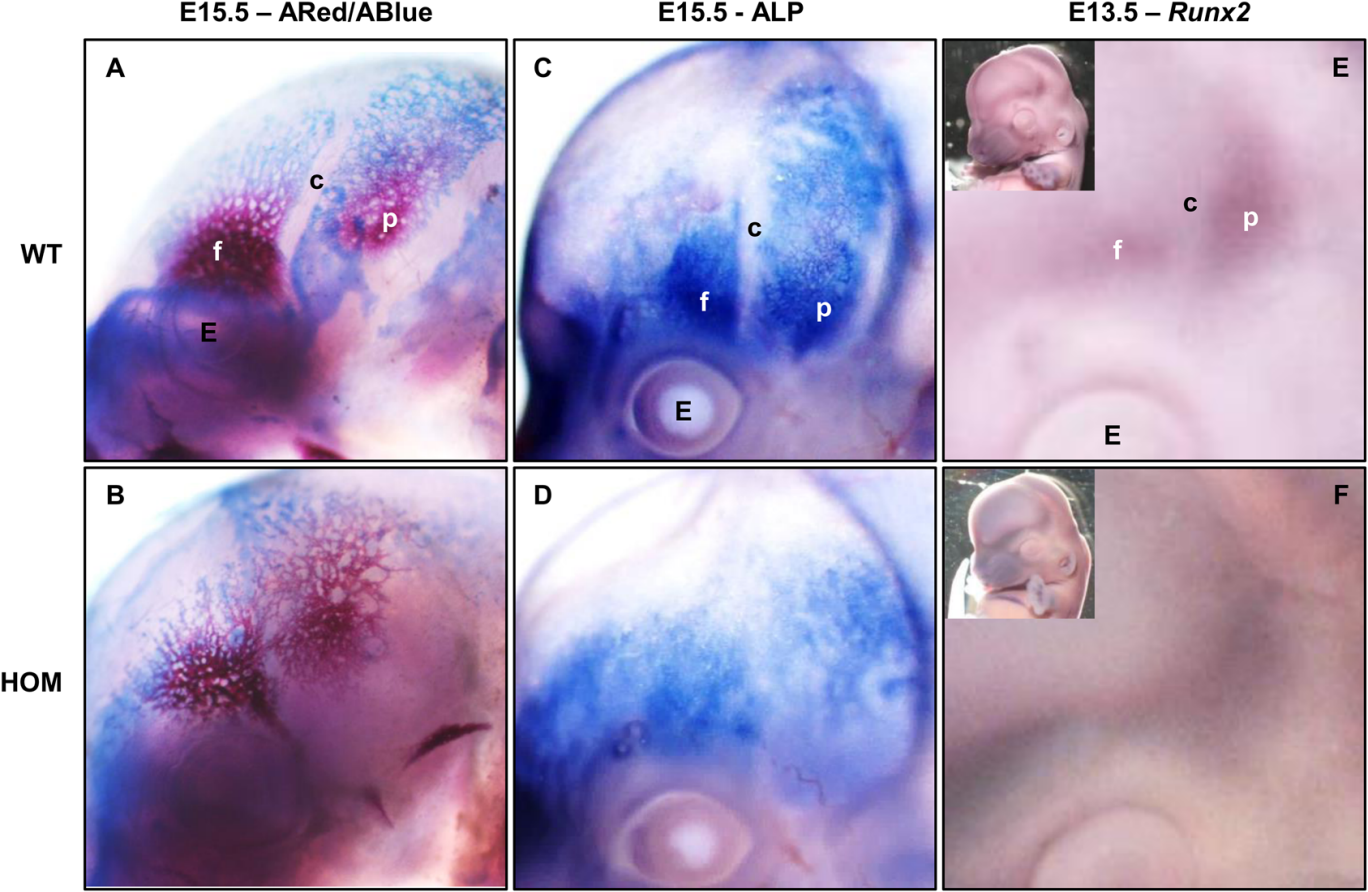
**Coronal suture formation in FGFR2-C342Y homozygous embryos.** (A,B) Skeletal staining of E15.5 embryos comparing WT (*n*=4) and homozygote (*n*=3) with red indicating bone and blue indicating cartilage. (C,D) Alkaline phosphatase staining of calvarial bones at E15.5 show the lack of a coronal suture at pre-ossification stages in HOM mutant embryos (*n*=4) compared to WT controls (*n*=4). (E,F) Expression of *Runx2* at E13.5 shows continuous expression from the frontal to the parietal bone in the HOM mutant (*n*=2). WT control (*n*=2) shows expression in frontal and parietal bones separately. E, eye; f, frontal bone; p, parietal bone; c, coronal suture.

While FGFR2 mutation affects *Sox9* expression in chondrocytic skeletal precursors, calvarial bones are intramembranous without a cartilaginous intermediate stage. Skeletal staining at E15.5 of WT and HOM embryos shows an apparent merging of the frontal and parietal ossification centres, suggesting a lack of sutural mesenchyme (Fig. 6A,B). When analysing the coronal suture in homozygous mutants using alkaline phosphatase (ALP) staining at E15.5 shows a continuous area of osteoblast activity while no suture can be observed (Fig. 6C,D). At this stage, heterozygous mutants are indistinguishable from wild-type controls (data not shown). This corresponds to the expression domain of *Runx2* at E13.5, where the suture can be seen between the frontal and parietal bones in controls (Fig. 6E,F); instead homozygous mutants show a continuous expression domain indicating failure of the coronal suture to form.

## DISCUSSION

Our knowledge of the normal development of the mammalian skeleton and the mechanism behind the pathogenesis of associated craniofacial birth defects remains incomplete. In this study, we identified novel phenotypic features in a mouse model for Crouzon syndrome and were able to show that FGFR2 plays a role in the early patterning of skeletal tissues by regulating the process of mesenchymal condensation.

### Phenotypic features associated with homozygous mutation of FGFR2

Studying the homozygous mouse mutants in more detail at embryonic stages has allowed us to characterise a more comprehensive phenotypic spectrum that better reflects the clinical spectrum of human Crouzon-Pfeiffer syndrome (CPS). For example, tracheal cartilaginous sleeve (TCS) (Scheid et al., 2002) and butterfly vertebrae (Anderson et al., 1997) are only rarely found in severe cases of human CPS, but have complete penetrance in mice homozygous for the p.C342Y mutation in FGFR2. It appears that the clinical spectrum in patients with dominant (heterozygous) mutations in *FGFR2* is reflected by the complete range (i.e. wild-type→heterozygote→homozygote) in the mouse model for Crouzon syndrome. This indicates a dose-dependent effect, which may be reflected in heterozygous patients due to their different (epi-)genetic background. In this light, it is interesting to consider the phenotype of the recently reported *Fgfr2*^C342Y/−^ hemizygous mutant (Pfaff et al., 2016). Here, the hemizygotes display a more severe form of craniosynostosis and midfacial hypoplasia, but don't have cleft palate, indicating a phenotypic severity between the C342Y heterozygote and homozygote. Despite the more severe phenotype in homozygotes, we have not been able to observe any of the rare limb defects (i.e. broad thumb/toe and/or radio-ulnar synostosis of the elbow) associated with CPS. This confirms the resistance of the mouse to FGFR2-related limb defects, similar to mouse models for Apert syndrome (AS) that have normal limbs (Wang et al., 2005).

The combined observation of cleft palate and calvarial hypoplasia in homozygotes resembles some of the clinical features of human AS (Kreiborg et al., 1993; Kreiborg and Cohen, 1992). If a dose-dependent effect is responsible for the exaggerated phenotype in the homozygous Crouzon mice, it is tempting to speculate that the AS mutations are more activating than the CPS mutations, to the point where a heterozygous AS mutation equals a homozygous CPS mutation. However, calvarial hypoplasia has not been observed in the Apert mouse model (Wang et al., 2005). In contrast, the craniofacial phenotype of the *Fgfr2*^W290R^ mouse model does include calvarial hypoplasia/delayed ossification (Gong, 2012). Thus, even though there is evidence that AS and CPS mutations activate FGFR2 differently (Plotnikov et al., 2000), with different effect (Mansukhani et al., 2000), the mechanism behind these differences and their impact on genotype-phenotype correlation remains unresolved.

Our data on the tracheal phenotype also shows that heterozygous mutant animals (which thrive and are fertile) display a less severe, occult form of TCS with partial fusion of mainly proximal tracheal rings, notably the fusion of the cricoid cartilage to the first tracheal cartilage ring. These phenotypic observations supply a possible alternative cause as one of the mechanisms for obstructive sleep apnoea which can be observed in up to 50% of CPS patients and has been suggested to be the results of either midfacial hypoplasia or raised intracranial pressure (Bannink et al., 2010). A recent paper describes TCS in five out of nine patients with a mutation at site C342 (Wenger et al., 2016), supporting our hypothesis that homozygous FGFR2-C342Y mice mimic severe features associated with heterozygote CPS patients and that screening for this condition in CPS patients should be considered.

Abnormalities in parts of the endochondral skull base in homozygous mutant animals include fusion of the tympanic bulla to the lateral edge of the cranial base. FGF receptor signalling and expression of *Fgfr2* has been reported to play an important role during the development of the inner ear (Lysaght et al., 2014; Wright et al., 2003). Also, inner and middle ear malformations have been described in CPS and AS patients (Orvidas et al., 1999; Zhou et al., 2009). We speculate that aspects of the malformation seen in homozygous mice may contribute to the high incidence of hearing loss reported in FGFR2-related cases of syndromic craniosynostosis, including 74% of CPS patients (Agochukwu et al., 2014) and warrants a more detailed investigation.

Neural tube defects have been described in rare cases of AS (Breik et al., 2016) and non-syndromic craniosynostosis (Borkar et al., 2011), but not in CPS. Despite this, a neural tube defect in *Fgfr2* mutant mice is not entirely unexpected as a role for FGF signalling through FGFR2 during neural tube development has been documented previously (Walshe and Mason, 2000; Wright and Mansour, 2003). Our own data confirms the expression of *Fgfr2* in the ectoderm of the neural plate during formation of the neural tube in the mouse (data not shown). The eye lid phenotype in the most severely affected homozygous mutants with exencephaly is intriguing because of the reported role of FGFR2 signalling (Li et al., 2001) and its association with neural tube defects (Yu et al., 2006), despite no reports of this clinical feature in CPS patients.

### Dysregulation of FGFR2 signalling disrupts *Sox9* expression in prechondrocytes and causes mesenchymal condensation defects

The role of SOX9 in the development of the cartilaginous skeleton is well documented. First, the *Sox9* mouse knockout displays hypoplastic cartilage, demonstrating an essential role for SOX9 in the establishment of the initial chondrocyte progenitor population in mesenchymal condensations (Bi et al., 1999). Second, it has been shown that FGF receptor signalling induces SOX9 expression in chondrocytes *in vitro* (Murakami et al., 2000b). Our data shows that a p.C342Y mutation in FGFR2 causes an increased and ectopic expression of the chondrocytic progenitor marker *Sox9* in precursor cells of the developing cartilage at E12.5. This is in contrast to a previous study on the Crouzon mouse model, where no differences in *Sox9* expression were revealed (Eswarakumar et al., 2004). It is possible that Eswarakumar et al. have looked at different cartilage primordia (i.e. humerus) and/or at different stages of embryonic development. However, a relationship between *Fgfr2* and *Sox9* has been reported in the embryonic development of the pancreas (Seymour et al., 2012b) and the testis (Kim et al., 2007), while activation of FGFR2 through the p.C278F mutation has been associated with induction of chondrogenesis *in vitro* (Petiot et al., 2002). Thus, the observation that mutation of FGFR2 affects the cartilaginous aspects of skeletal development as well as the intramembranous ones is not entirely unexpected.

Next to SOX9, an important role during tracheal development has been assigned to FGF10 and SHH (Park et al., 2010; Tiozzo et al., 2009). Indeed, a relationship between FGF signalling and periodic expression of *Shh* in tracheal epithelium has been established as the mechanism behind the formation of distinct cartilage rings (Sala et al., 2011). Sala et al*.* have shown that overexpression of *Shh* in the ventral epithelium leads to an upregulation of *Sox9* and COL2A1, but prevents mesenchymal condensation into distinct cartilage primordia. This is similar to what we have observed in our homozygous Crouzon mutants. Tracheae show an increased expression of *Shh* in the ventral epithelium and impaired mesenchymal condensation. Loss of periodic expression of *Shh* can be caused by either an increase or decrease of mesenchymal FGF10 according to Sala et al., which is reminiscent of our mutants where a decrease in the ventral mesenchyme is observed. How the intra epithelial-mesenchymal gap plays a role in the failure of mesenchymal condensation remains unclear, but it is tempting to speculate that the abnormal morphology impacts on the ability of epithelial SHH to signal to the neighbouring mesenchyme and/or for FGF10 to signal to the neighbouring epithelium. The FGFR2 isoform affected by the p.C342Y mutation (IIIc) is expressed in the mesenchyme and it has been reported that FGFR2c regulates the expression of *Fgf10* (Colvin et al., 2001). Taken together, our data suggests that mutant FGFR2c in the mesenchyme disrupts *Fgf10* expression. As FGF10 acts on neighbouring epithelial cells, this leads to the loss of segmented expression of *Shh* in the tracheal epithelium, which in turn perturbs correct mesenchymal condensation of the chondrogenic mesenchyme. Tracheal and cricoid cartilage are derived from the splanchnic mesoderm, the ventral layer of the lateral mesoderm. It is defined by the expression of *Foxf1*, which has been reported to be controlled by SHH (Mahlapuu et al., 2001). Based on cartilage staining with Alcian Blue (Fig. 3) and expression analysis of Sox9 (Fig. 4), the phenotypic defects observed in the upper airways of the Crouzon mouse appear to be restricted to the cricoid and trachea, with a clear proximal-distal range of decreasing severity, a possible role for FOXF1 can not be excluded and warrants further investigation.

### FGFR2-C342Y homozygous mutants do not form a coronal suture

Apart from its previously described role in osteogenic differentiation in the formation of cranial sutures, data from this study suggest that FGFR2 – via the regulation of *Sox9* expression – plays a role in the mesenchymal condensation of cartilage and cartilage precursors of endochondral bone. SOX9 has previously been found to control patterning of the posterior frontal suture (Sahar et al., 2005). When analysing the coronal suture in the *Fgfr2*^C342Y^ mouse model, we did not detect any *Sox9* expression at embryonic stages between E12.5 and E18.5 (data not shown). However, we did find that in homozygous mutants at E15.5, the suture was not visible when staining with alkaline phosphatase, a marker for mature osteoblasts. Furthermore, we did not detect a suture when performing whole mount *in situ* hybridisation using a probe against *Runx2*. The lack of a coronal suture at this stage implies that the coronal suture was never formed in these mutants. Deckelbaum et al. have shown that the coronal suture forms at the supraorbital region between E11.0 and E13.5 (Deckelbaum et al., 2012). They also identified a role for EN1 in regulating the osteogenic potential of sutural mesenchyme via FGFR2 signalling. Together, this suggests that activation of FGFR2 by the p.C342Y mutation in the mouse not only affects the differentiation of sutural mesenchyme, leading to a prematurely ossified suture resulting in synostosis in heterozygous mutants, but also plays a role in the patterning of the coronal suture at earlier stages of development, leading to the coronal suture failing to form in homozygous mutants. Whether FGFR2 plays a role in the condensation of intramembranous bone is the subject of further investigation. Data from the tracheal cartilage indicates that the synostosis phenotype is likely to be a failure of correct mesenchymal condensation, and that this process is regulated by the organisation of the extracellular matrix.

### Summary

We report here a comprehensive phenotypic analysis of the previously described Crouzon mouse model (Eswarakumar et al., 2004). Detailed analyses of the effects of the p.C342Y mutation in homozygous animals at embryonic stages have identified an expanded phenotypic spectrum that mimics the severe end of the clinical spectrum seen in heterozygous CPS patients. We find that the homozygous mutants represent the most severe end of the phenotypic spectrum, which has allowed us to study the causative molecular events and improve the understanding of the pathogenesis of Crouzon syndrome and related craniofacial birth defects caused by mutation of FGFR2. Some of the features that form part of the CPS phenotypic spectrum can be observed at embryonic stages before either chondrogenic or osteogenic differentiation. These results suggest that FGFR2 is involved in the early patterning as well as the maturation of bone and cartilage. Our data suggest that FGFR2 is involved in the process of mesenchymal condensation that determines the shape and size of mesenchyme-derived tissues like bone and cartilage. Further study of the mechanisms underlying the role of FGF signalling during mesenchymal condensation will contribute to an improved understanding of the pathogenesis of craniofacial birth defects and present an opportunity to explore clinical translation.

## MATERIALS AND METHODS

### Mice

Crouzon mice (*Fgfr2*^tm4Lni^, MGI number: 3053095) were re-derived through the European Mouse Mutant Archive (EMMA) at MRC Harwell (CD1-FGFR2c342y, number EM02488) and the alias *Fgfr2*^C342Y^ is used throughout this paper. The mice were originally described by Eswarakumar et al. (2004). Mice were maintained as heterozygous breeding pairs in the animal care facilities of UCL Biological Services. To prevent malocclusion in mutants due to midfacial hypoplasia, animals were provided with wet diet, sunflower seeds and wooden blocks for chewing. When necessary teeth were clipped using appropriate scissors. All timed matings were set up in the early evening and checked early morning. Litters/embryos were genotyped for heterozygous or homozygous presence of the C342Y mutant allele by standard PCR protocols. Primer sequences are available on request. Comparative analyses described in this paper using non-sexed mouse embryos have been performed with at least three littermates in each arm (i.e. WT, HET, HOM). All animal procedures were performed in accordance with the UK Animals (Scientific Procedures) Act 1986 (Project License number 70/7194).

### Histology

For skeletal staining, animals were euthanised by exposure to carbon dioxide gas in a rising concentration, fixed in 90% ethanol, then skinned and eviscerated. Staining with Alcian Blue (0.05%) was performed in 70% ethanol with 20% acetic acid, followed by staining with Alizarin Red (0.15%) in 1% KOH. Soft tissue was cleared with 1% KOH with 20% glycerol and skeletons were stored in 80% glycerol. For whole-mount alkaline phosphatase assay, embryos were dissected by removing the head, cutting it mid-sagittally and removing the brain, followed by fixation in 4% paraformaldehyde. Following fixation, embryos were washed in NTMT solution (0.1 M NaCl; 0.1 M Tris-HCl; 0.05 M MgCl2; 0.1% Tween 20) followed by staining with NBT-BCIP (18.8 mg/ml nitro-blue tetrazolium chloride; 9.4 mg/ml 5-bromo-4-chloro-3-indolylphosphate toluidine salt in 67% DMSO) in NTMT solution until the desired level of staining was reached. Embryos were washed and stored in PBS. Whole-mount-stained embryos were visualised using a microscope (Steni SV6 Zeiss) and attached camera (Leica DFC490). Histological staining of paraffin-embedded embryos, sectioned at 8 µm, was performed using standard staining protocols for Haematoxylin, Eosin, Alcian Blue and Nuclear Fast Red.

### *In situ* hybridisation

Expression analysis of *Sox9*, *Shh*, *Fgf10* and *Runx2* were performed using standard *in situ* hybridisation protocols using probes kindly provided by the Lovell-Badge laboratory (*Sox9*; The Francis Crick Institute), Greene laboratory (*Shh* and *Fgf10*; Institute of Child Health) and Ferretti laboratory (*Runx2*; Institute of Child Health).

### Immunohistochemistry

Protein expression analysis of type II collagen (COL2A1) was performed with a monoclonal antibody (Abcam number ab3092) using standard immunohistochemistry protocols. The antigen retrieval step was done using trypsin (0.5%) and calcium chloride (1%) at 37°C for 10 min and the dilution used was 1:100.
